# Identification of viral genes involved in pepper mottle virus replication and symptom development in *Nicotiana benthamiana*

**DOI:** 10.3389/fpls.2022.1048074

**Published:** 2022-10-28

**Authors:** Miao Fang, Jisuk Yu, Hae-Ryun Kwak, Kook-Hyung Kim

**Affiliations:** ^1^ Department of Agricultural Biotechnology, Seoul National University, Seoul, South Korea; ^2^ Plant Genomics and Breeding Institute, Seoul National University, Seoul, South Korea; ^3^ Crop Protection Division, National Institute of Agricultural Sciences, Rural Development Administration, Wanju, South Korea; ^4^ Research Institute of Agriculture and Life Sciences, Seoul National University, Seoul, South Korea

**Keywords:** pepper mottle virus, HC-Pro, virus-host interaction, viral symptom determinant, *Nicotiana benthamiana*

## Abstract

Pepper mottle virus (PepMoV) infects primarily *Capsicum* species, including pepper and bell pepper which are important vegetable and spice crops in Korea. We have previously collected 13 PepMoV isolates from nine regions comprising five provinces, causing different symptoms on inoculated indicator host plants in Korea. To further identify the responsible symptom determinant(s) and explore viral protein functions of PepMoV, two out of 13 isolates, including 134 and 205136, were used in this study. Isolate 134 causes necrosis and yellowing, while 205136 causes severe mottle and yellowing symptoms on *Nicotiana benthamiana*. All chimeric and site-directed mutants contain the PepMoV 134 genome as a backbone with specific regions switched for those from counterparts of PepMoV 205136. Effects of all mutants compared with 134 after inoculation onto *N. benthamiana* by agroinfiltration. Results from our study provide direct evidence that the helper component-proteinase (HC-Pro) and the nuclear inclusion protein b (NIb)-coat protein (CP) regions are involved in virus accumulation and symptom determinants. In addition, we mapped to amino acid residues tyrosine, glycine, and leucine at position 360, 385, and 527, respectively, in the HC-Pro region participate in faster viral accumulation or movement in the plant. The residue valine at position 2773 of NIb plays an essential role in isolate 134 symptom development. As part of this study, we seek to gain insight into viral factors involved in the PepMoV infection cycle and a better understanding of plant-virus interactions. These findings complement the insufficiency of the gene function study of the PepMoV virus and provide a novel perspective for the protein function study of the *Potyvirus*.

## Introduction

*Pepper mottle virus* (PepMoV), a member of the *Potyvirus*, the largest genus of plant RNA virus, causes significant losses of economically important crops (Rojas et al., 1997; Kim et al., 2008). PepMoV forms a flexuous rod-shaped virion with a length of 730 nm and a diameter of 12 nm containing a single-stranded plus sense RNA genome of 9.7 kb in length (Warren and Murphy, 2003; Kim et al., 2008). Viral genomic RNA, covalently linked to a viral-encoded protein (VPg) at the 5’ end and containing a polyadenylated tail at the 3’ end, encodes a large polyprotein cleaved by three virus-specific proteases to yield 11 mature proteins (Chung et al., 2008). As in all potyviruses, PepMoV encodes two polyproteins, a large polyprotein of approximately 3,068 amino acid residues and the shorter one translated from a 2+ frameshifting of the P3 cistron, as a fusion to the amino (N)-terminal part of P3 (P3N-PIPO) (Quenouille et al., 2013).

PepMoV infects most *Capsicum* sp. and is transmitted by aphids in a non-persistent manner in fields (Han et al., 2006). Our laboratory has previously isolated 13 PepMoV isolates, which were collected from nine regions comprising five provinces in Korea (Kim et al., 2009). These 13 PepMoV isolates caused different symptoms on indicator host plants, i.e., *Nicotiana tabacum* cv. Xanthi-nc and *N. benthamiana* (Kim et al., 2009). Among them, isolate 134 caused necrosis and yellowing symptoms in *N. benthamiana*, while isolate 205136 caused mild mottle symptoms in *N. tabacum* and severe mottling and yellowing symptoms in *N. benthamiana* (Kim et al., 2009).

Among research on identifying viral pathogenicity determinants, *Potato virus Y* (PVY, the type member of the genus *Potyvirus*) is one of the intensively studied viral pathogens (Scholthof et al., 2011; Quenouille et al., 2013). The helper component-proteinase (HC-Pro) is the critical protein encoded by potyviruses, characterized in detail. The HC-Pro intervenes in several steps of the virus replication cycle: 1) genome multiplication and replication at the single-cell level; 2) cell-to-cell and systemic movements (Kasschau et al., 1997; Rojas et al., 1997; Sáenz et al., 2002); and 3) symptom intensity (Shiboleth et al., 2007; Torres-Barceló et al., 2008; Yambao et al., 2008). HC-Pro can be schematically divided into three regions: an N-terminal region essential for the aphid transmission process, a C-terminal region harboring the proteinase activity and the suppression of plant defenses based on the RNA silencing machinery by binding small interfering RNAs (siRNAs), and a central region implicated in all other functions (Plisson et al., 2003; Lakatos et al., 2006; Shiboleth et al., 2007; Varrelmann et al., 2007). These various properties could account not only for the general involvement of the potyvirus HC-Pro in symptomatology and synergy with co-infecting viruses for symptom severity but also for its roles in virus multiplication and systemic movement.

The nuclear inclusion a (NIa) protease of potyviruses has been identified as a multiprotein that plays various roles in virus infection (Xiao et al., 2022). The tobacco etch virus (TEV) NIa has RNA binding activity; it can interact with NIb protein and contributes to viral accumulation (Schaad et al., 1996; Daròs and Carrington, 1997). NIa of papaya ringspot virus (PRSV) is involved in host specificity (Chen et al., 2008). The NIa of PepMoV has been reported as a pathogenicity determinant and contributed to releasing DNA methylation of *N. benthamiana* (Gong et al., 2020). The NIb protein has been shown to act as the RNA-dependent RNA polymerase (RdRp) involved in the replication of viral RNA (Hong and Hunt, 1996). The NIb protein of TEV possesses several functions, including RdRp and nuclear translocation activities (Li et al., 1997). In addition, PepMoV NIb functions as an elicitor for the potyvirus-resistant 4 (Pvr4) and Pvr9-mediated hypersensitive responses (Kim et al., 2015; Tran et al., 2015). NIb from several other closely related potyviruses also elicited a similar hypersensitive response (Tran et al., 2015). The P3 protein of turnip mosaic virus (TuMV), which is an influential pathogen of *Brassica* species and other crops worldwide (Walsh et al., 2002), has been identified as a symptom and avirulence determinant in Brassicas (Jenner et al., 2003). The viral RdRp or RNA replicase is expected to contribute to indirectly defining pathogenesis by affecting virus replication and thus virus accumulation (García and Pallas, 2015). Based on what has been discussed above with potyviruses, including PVY, TuMV, and TEV, the functions of their proteins might differ depending on the virus species; up to now, the protein functions of PepMoV remain largely unknown.

In terms of plant RNA viruses, *Potyvirus* is the largest genus; they have been well documented. However, those species’ viral protein functions differ from virus to virus. Research on PepMoV and its protein functions are largely unknown. In this study, we revealed the crucial region(s) of PepMoV for symptom development and systemic infection using a full-length infectious clone of PepMoV 134 expressing green fluorescent protein (GFP; pPepMoV-134:GFP) (Tran et al., 2019). Our results suggested that the HC-Pro is involved in PepMoV genome replication and systemic movement, the C-terminal region of the NIb plays a vital role in developing viral symptoms and multiplication. In addition, amino acid substitution mutations revealed that the valine residue at position 2773 in the C-terminal region of the NIb protein is critical in causing different symptoms without noticeably affecting the systemic movement of the virus. Taken together, our results adequately fill in the large gaps in PepMoV viral protein functions. Additionally, our findings provide insight into viral proteins implicated in the PepMoV infection cycle and a novel perspective for the biology study of potyvirus.

## Materials and methods

### Plant and virus sources

**Figure 1:**
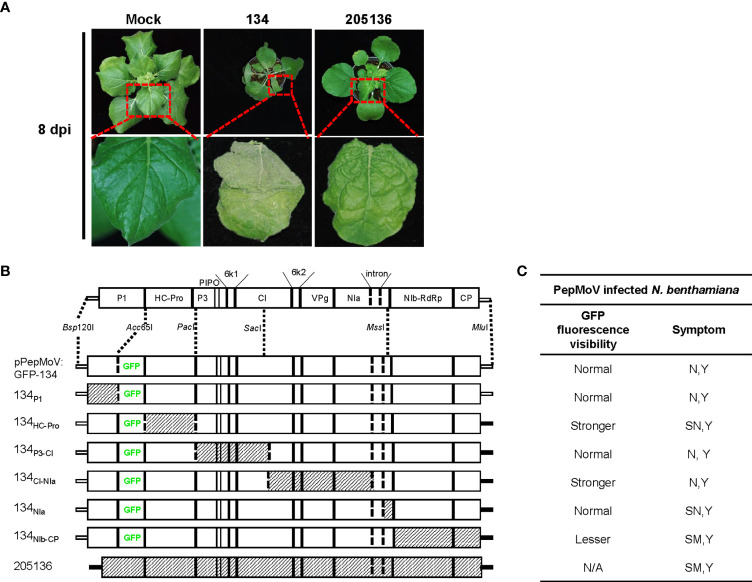
**(A)** Symptoms on *N. benthamiana* leaves infected with pepper mottle virus (PepMoV) isolates 134 and 205136. Results from upper systemic (non-inoculated) leave at 8 days post-inoculation (dpi) are shown. **(B)** Schematic representation of chimeric mutants of PepMoV isolates 134 and 205136. Chimeric mutants were constructed by substituting counterparts between 134 and 205136. pPepMoV : GFP-134 and 205136-derived regions are indicated by blank and hatched boxes, respectively. **(C)** Summary of 134 mutants shown in **Figures 2** and **3**. GFP fluorescence representing virus infection and symptoms of the other six chimeric viruses were compared with PepMoV isolate134. Viral accumulation was observed at 5 and 8 dpi, and symptom development was detected at 12 dpi. N, necrosis; Y, yellowing; SN, severe necrosis; SM, severe mottle; N/A, not available.

*N. benthamiana* was used as an indicator host plant in this study. *N. benthamiana* seedlings were selected for inoculation when plants were three weeks old. *N. benthamiana* plants were grown in a growth chamber at 25°C under a 16/8 h (light/dark) photoperiod. The PepMoV 134 and 2051356 isolates have been described and characterized previously (Kim et al., 2009). Infectious full-length cDNA clones of PepMoV 134 (pPepMoV: GFP-134) were previously constructed (Tran et al., 2019). Isolate 205136 was propagated in *N. benthamiana* plants with infected tissue and collected virus-infected systemic leaves after symptom observation (**Figure 1A**).

### Generation of chimeric and site-directed mutants

The complete genome sequence of isolate 205136 was divided into 6 fragments and exchanged individually with the corresponding pPepMoV : GFP-134 plasmid region. The main 11-kb section was divided using conserved restriction enzyme sites to make reciprocal exchanges between pPepMoV : GFP-134 and counterparts DNA fragments of isolate 205136 of the 1.8-kb *Bsp*120I- *Acc*65I, 4.2-kb *Acc*65I- *Pac*I, 2kb *Pac*I*-Sac*I, 2.8-kb *Sac*I- *Mss*I and 0.52-kb *Sac*I- *Mss*I and 2.5-kb *Mss*I- *Mlu*I fragments. Chimeras were constructed by exchanging counterparts between pPepMoV : GFP-134 and DNA fragment of isolate 205136 using available restriction enzyme sites common to the plasmid of pPepMoV : GFP-134 and DNA fragments of isolate. The used restriction enzyme sites in the PepMoV genome are shown in **Figure 1B**.

Amplification of DNA fragments to produce chimeras and amino acid substitution mutants was carried out by PCR with virus-specific primer pairs. To introduce site-directed mutagenesis (SDM) in pPepMoV : GFP-134, DNA fragments of isolate 205136 were amplified for 40 cycles using PfuUltra II Fusion HS DNA polymerase (Agilent Technologies, U.S.A.) and appropriate primer pairs: mutant4 I2374V Fw and mutant4 I2374V Rv primers for the mutant4 I2374V, mutant4 V2773D Fw and mutant4 V2773D Rv primers for the mutant4 V2773D, mutant4 T2789A Fw and mutant4 T2789A Rv primers for the mutant4 T2789A, mutant4 T2805A Fw and mutant4 T2805A Rv primers for the mutant4 T2805A. The PCR products were purified through a DNA purification kit (NucleoSpin Gel and PCR clean-up Kit, MACHEREY-NAGEL, Germany). The mutagenized PCR fragments, amplified to contain the region from *Mss*I to *Mlu*I sites, were digested with *Mss*I and *Mlu*I and inserted into pPepMoV : GFP-134, which was digested with *Mss*I and *Mlu*I. The sequences of all constructed chimeras and mutants were validated by DNA sequencing. All primers for constructing chimeras and amino acid substitution mutants are listed in **Supplementary Table 1**.

### Plant inoculation and virus assessment

To evaluate the effects of all modified pPepMoV : GFP-134 on *N. benthamiana*, all expression clones were prepared using a plasmid purification kit (MACHEREY-NAGEL, Germany), including pPepMoV : GFP-134, then transformed into *Agrobacterium tumefaciens* strain GV3101 by electroporation device (ECM 830, BTX, USA). The presence of transformants in the *Agrobacterium* was detected by PCR with specific primers. The agroinfiltration was conducted as described previously (Yoon et al., 2021). The optical density of each bacterial suspension was measured at OD_600_ with a UV/visible spectrometer (Ultrospec 3100 Pro, Biochrom, Cambridge, England). Then the suspensions were diluted to the OD_600_ to 0.5. Fully expanded leaves of three-week-old plants were infiltrated by syringes to their backsides. The treated leaves were photographed with a digital camera (Nikon7200, Tokyo, Japan). Necrosis symptom differentiation was assessed visually at 12 dpi. PepMoV local and systemic movements on inoculated plants were verified by observing GFP expression under UV light at 5 and 8 dpi.

### Protoplast isolations

For protoplast isolation, inoculated with wild-type 134 and chimeric virus suspensions (OD_600 =_ 0.5) onto plants by agroinfiltration and then incubated for 2 hours in a growth chamber at 25°C. Protoplasts were isolated from infected sliced leaves (remove the petiole and midrib) and digested with 10ml enzyme solution (cellulose enzyme solution including 1% Cellulase R10 (Yakult), 0.5% Macerozyme R10 (Yakult), 0.45 M Mannitol and 20 mM MES (pH 5.7) for 14 to16 hours at 25°C, 10 rpm shaking for 10 mins before collection. And then, dilute the enzyme/protoplast solution with an equal volume of W5 washing solution. Protoplasts were collected using filtration followed by centrifugation at 750 rpm 4°C for 3 mins in a swinging-bucket rotor. Rewash protoplast with 2 ml of W5 washing buffer, centrifuge at 750 rpm 4°C for 3 min, and then resuspend in 1 ml of WI incubation solution.

### RNA extraction and cDNA synthesis

Total RNAs were isolated from healthy and virus-infected *N. benthamiana* leaves using RNAiSO Plus reagent (TaKaRa) according to the manufacturer’s instructions. The viral RNA extraction protocol of protoplast is referred from a previous study (Fabian and Andrew White, 2007).

Equal amounts of total RNAs (1 µg) were used for cDNA synthesis using M-MLV reverse transcriptase (Promega, USA) and oligo (dT_15_) primer (Bioneer). cDNAs were used for virus detection and quantification of viral RNA accumulation for further experiments. PepMoV was detected by PCR using virus-specific primer pair (**Supplementary Table 1**).

### RT-qPCR

RT-qPCR reactions were conducted using IQ™ SYBR Green Supermix (Bio-Rad, USA) based on manual instruction in a CFX 384 Real-Time PCR detection system (Bio-Rad, USA). The RT-qPCR analysis was conducted as previously reported (Widyasari et al., 2022). The endogenous gene actin was used as a reference gene to normalize the qPCR results. Melting-curve analysis was carried out using the Bio-Rad CFX manager v.1.6.541.1028 software. Experiments were repeated three times with at least three replicate plants in three independent experiments. The primer sets used for qPCR are shown in **Supplementary Table 1**.

### Statistical analysis

Experiments were conducted at least three times with three individual plants (biological replicates) in each experiment. Statistical analysis was performed using IBM SPSS statistics 26 for Windows software. The data were subjected to a one-way analysis of variance (ANOVA). The means of values were compared using Duncan’s least significant range test (p<0.05). Graphs were generated using GraphPad Prism.

## Results

### Symptom observations of chimeric mutants of PepMoV isolate 134 and 205136

The reported 13 PepMoV isolates caused different symptoms in *N. benthamiana*, respectively. In this work, we selected two isolates, 134 and 205136, which displayed visible and significantly different symptoms (**Figure 1A**). Based on a previous study (Tran et al., 2019), we used a full-length infectious clone of isolate 134 (pPepMoV : GFP-134) to investigate the differences in symptoms development caused by 134 and 205136 in detail; we constructed six PepMoV : GFP-134 chimeric clones whose specific viral region was precisely replaced with corresponding regions of PepMoV 205136 isolate. We designed chimeric regions based on available restriction enzyme sites of PepMoV-134 and 205136 viral cDNA for exchanging and generating chimeric clones from pPepMoV : GFP-134 as a backbone vector (**Figure 1B**). In addition, all chimeric mutants contained GFP downstream of the P1 region to evaluate GFP-expressing PepMoV in plants. A scale card was developed to classify mutants’ differences in GFP fluorescence expression and symptom development (**Supplementary Figure 1**). GFP fluorescence expression and symptom development for all mutants are summarized in **Figure 1C**.

**Figure 2:**
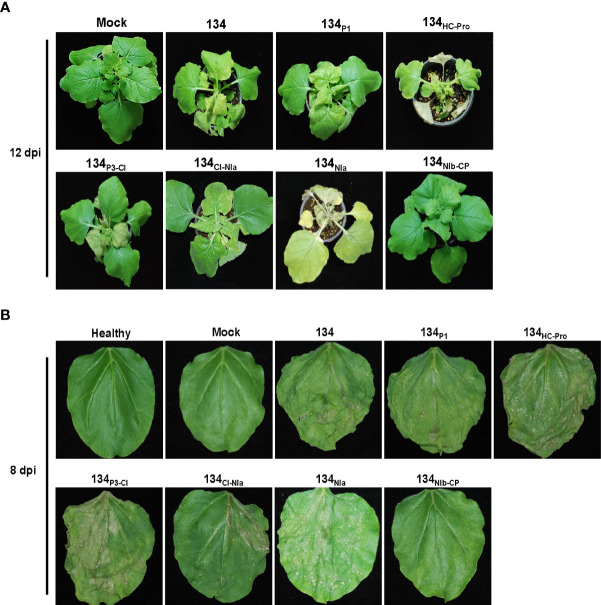
Symptom development of systemic and infected leaves on *N. benthamiana* plants caused by 134 and the other mutants. **(A)** Symptoms of 134 and chimeric mutants at 12 dpi. **(B)** Passage experiments with sap extracts of 134 and six chimeric mutant viruses inoculated onto *N. benthamiana*.

Symptom development of pPepMoV : GFP-134 and the chimeric mutants were monitored at 12 dpi. In the previous study, we confirmed consistent results in pPepMoV : GFP-134 infected *N. benthamiana* plants. pPepMoV : GFP-134 caused necrotic and yellowing symptoms on systemic leaves. Most symptoms appeared at 12 dpi in all tested plants except 134_NIb-CP_ infected plants. 134_NIb-CP_ was unable to display necrosis symptoms (**Figure 2A**). 134_NIb-CP_ infected plants did not show necrosis symptoms at 16 dpi (**Supplementary Figure 2**). Similar to isolate 134 (pPepMoV : GFP-134), 134_P1,_ 134_P3-CI_, and 134_CI-NIa_ induced necrosis symptoms on local and systemic leaves. In contrast, 134_HC-Pro_ and 134_NIa_ caused more severe necrotic symptoms when compared with 134. These results indicated that NIb and/or CP proteins of PepMoV might be responsible for causing different symptoms of PepMoV. Additionally, the HC-Pro and C-terminal of NIa may participate in causing necrosis symptoms of PepMoV.

Serial passaging experiments were performed by sap inoculation to determine the stability of the altered symptomatology for six mutants. Inoculation of sap extracts from 134_P1,_ 134_HC-Pro_, 134_P3-CI_, 134_CI-NIa_, and 134_NIa_ induced consistent necrotic symptoms. However, 134_NIb-CP_ caused only yellowing and mottle symptoms compared to 134. As a result, most chimeric mutants sustained stable viral infectivity in the plant host (**Figure 2B**).

### GFP fluorescence representing chimeric virus infections in *N. benthamiana*

**Figure 3:**
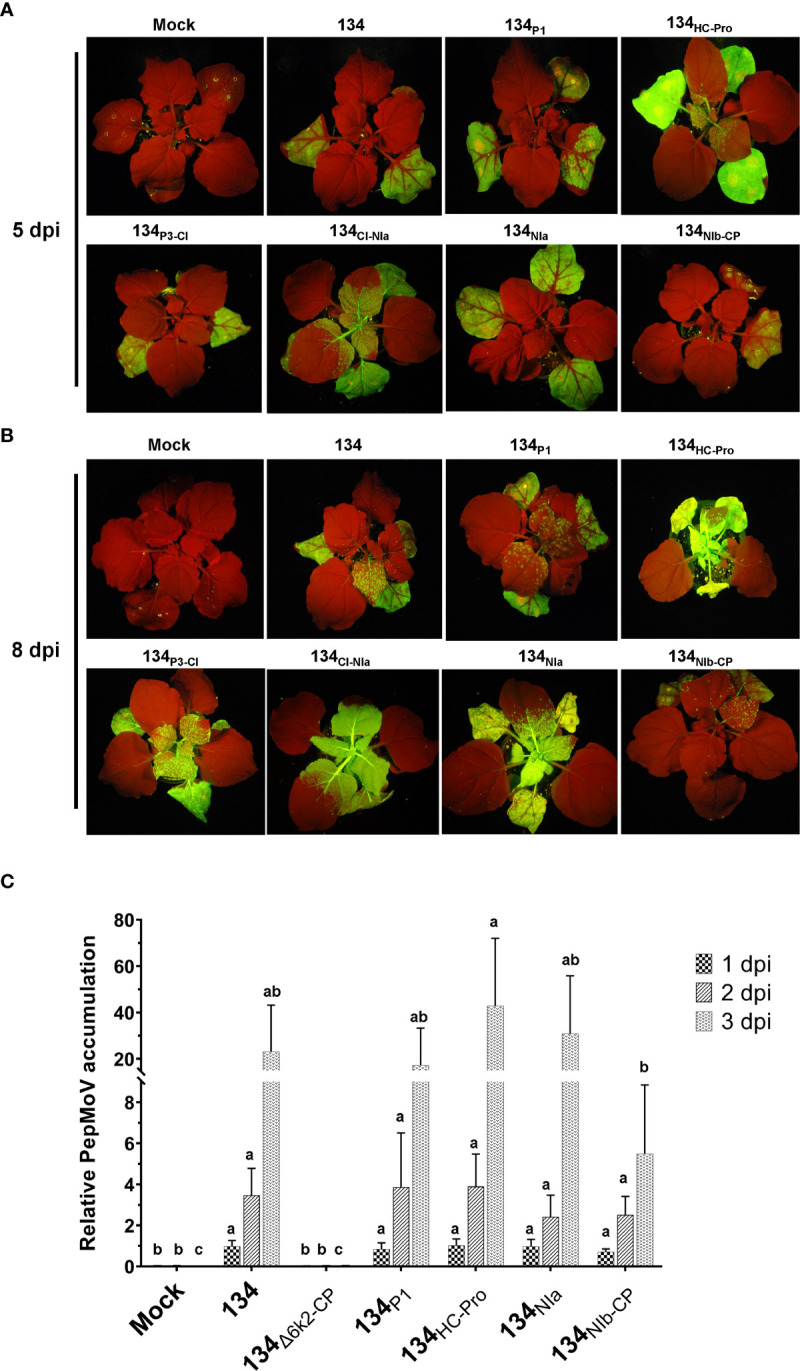
GFP fluorescence representing chimeric viruses infection and symptom development of parental and mutant viruses on local and systemic leaves. PepMoV-134 and chimeric mutants were inoculated onto *N. benthamiana* plants by agro-infiltration. The plant age used for inoculation is around 3 weeks old. Differences in virus replication among 134 and mutant viruses were monitored from 5 to 12 days post-inoculation. **(A)** Results of inoculated leaves at 5 days post-inoculated (dpi). **(B)** Results of systemic leaves at 8 dpi. **(C)** Virus accumulation at 1 dpi, 2 dpi, and 3 dpi, respectively. Mean values with different letters above the bars indicate significant differences at *P* < 0.05 according to a one-way ANOVA and Duncan’s least significant range test.

The GFP-expressing virus accumulation of chimeric mutant viruses was monitored under UV light (**Figure 3A**). At 5 dpi, inoculated leaves of the 134_P1,_ 134_P3-CI_, and 134_NIa_ displayed no noticeable difference in GFP intensity compared with 134. The 134_HC-Pro_ and 134_CI-NIa_ produced more vigorous GFP intensity on the local leaves and faster systemic movement to upper leaves, indicating that PepMoV accumulation in those mutants was higher than that of 134. On the contrary, the 134_NIb-CP_ induced distinct lesser viral accumulation on inoculated leaves than 134. At 8 dpi, GFP expression of 134- and chimeric viruses-inoculated plants moved to the upper non-inoculated leaves (**Figure 3B**). The 134_HC-Pro_ and 134_CI-NIa_ infected plants showed vigorous GFP intensity, and 134_NIb-CP_ infected plants showed weak GFP expression in systemic leaves.

To determine whether the symptoms caused by chimeric mutants correlated with viral RNA accumulation, we conducted quantitative real-time PCR (RT-qPCR). After the 134 and four selected chimeric mutants were inoculated onto *N. benthamiana*, total RNAs were extracted from local inoculated leaves at 1 to 3 dpi. We also included another mutant (134_Δ6k2-CP_) of pPepMoV : GFP-134 as a negative control, which did not contain the 6K2 to CP region in the 134 viral genome and lacks replication activity since it does not have RdRp. At the early viral replication stage, no significant difference was observed among 134 and chimeric mutants at 1 dpi based on RT-qPCR analysis (**Figure 3C**). Although the mutant 134_HC-Pro_ showed increased viral RNA accumulation (185%) and the 134_NIb-CP_ showed reduced viral RNA accumulation (23%) at 3 dpi compared to 134 these are consistent results with their phenotype on plants, these changes were insignificant due to high experiment deviations (**Figure 3**). In the case of 134_NIa_ and 134_P1_, viral RNA accumulation in those mutants showed similar accumulation levels compared to 134. This result suggests that the NIb-CP region of 205136 would adversely impact RNA accumulation, cell-to-cell and systemic movement of isolate 134. In contrast, the HC-Pro region might play an imperative role in virus replication and systemic movement (**Figures 3B, C**).

### Evaluation of PepMoV replication on chimeric mutants at a single cell

**Figure 4:**
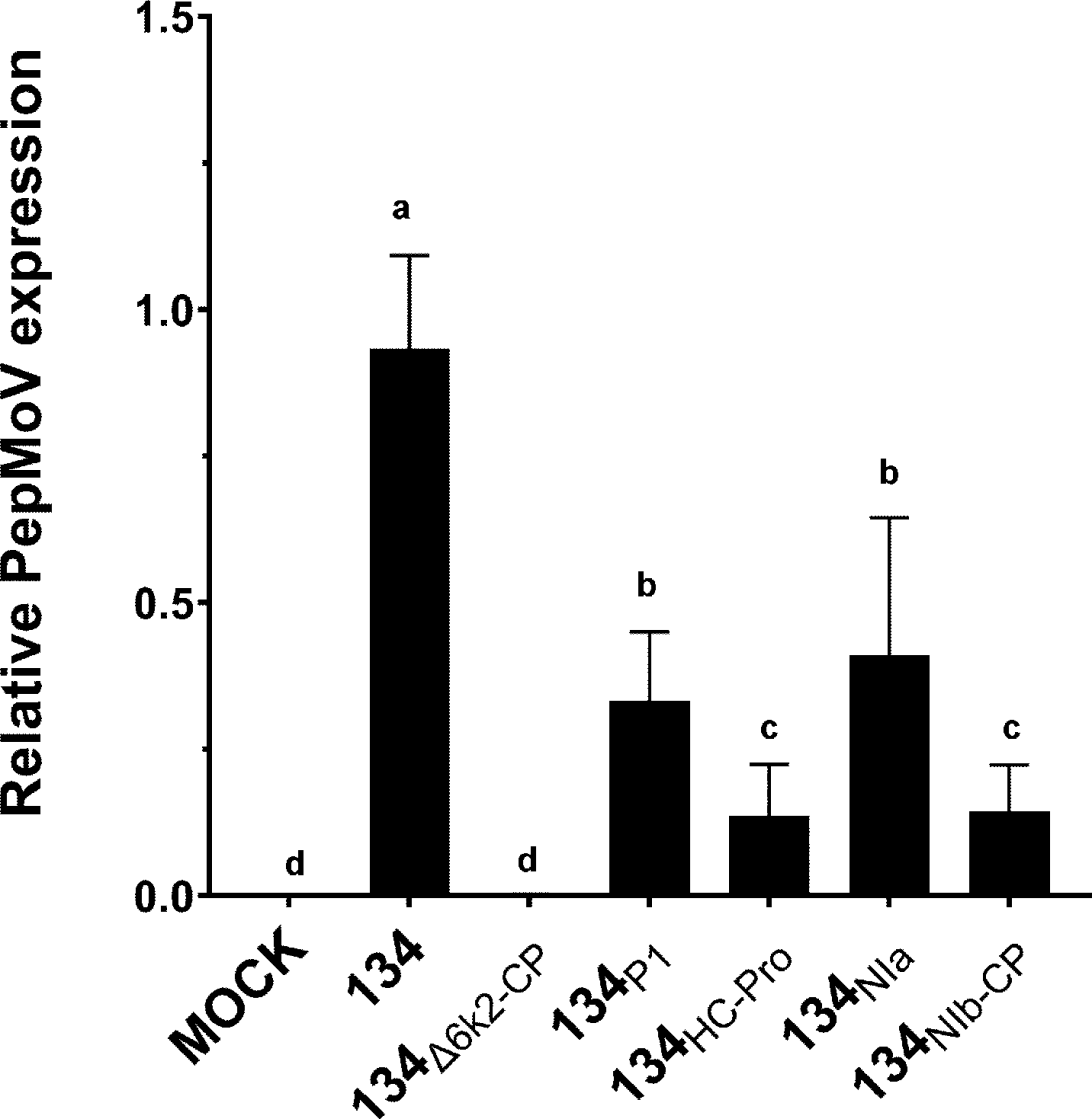
Quantification of protoplast viral accumulation at 1 dpi. Mean values with different letters indicate significant differences (*P* < 0.05) between 134 and chimeric mutants according to a one-way ANOVA and Duncan’s least significant range test (*P* < 0.05).

Since the RT-qPCR results indicated no significant differences between 134 and chimeric viruses at 1dpi (**Figure 3C**), we isolated protoplasts from those mutant-infected plants to identify the conditions of viral accumulation differentiation. As a result, on a single cellular level, viral RNA accumulation of all mutants was significantly lower than 134 (**Figure 4**). When the viruses were present in the cells that were initially infected, there were no distinguishing characteristics among the mutants; however, as the viruses moved from cell to cell, the impacts of chimeric mutants became apparent.

### Generation of amino acid substitution clones of 134_HC-Pro_

**Figure 5:**
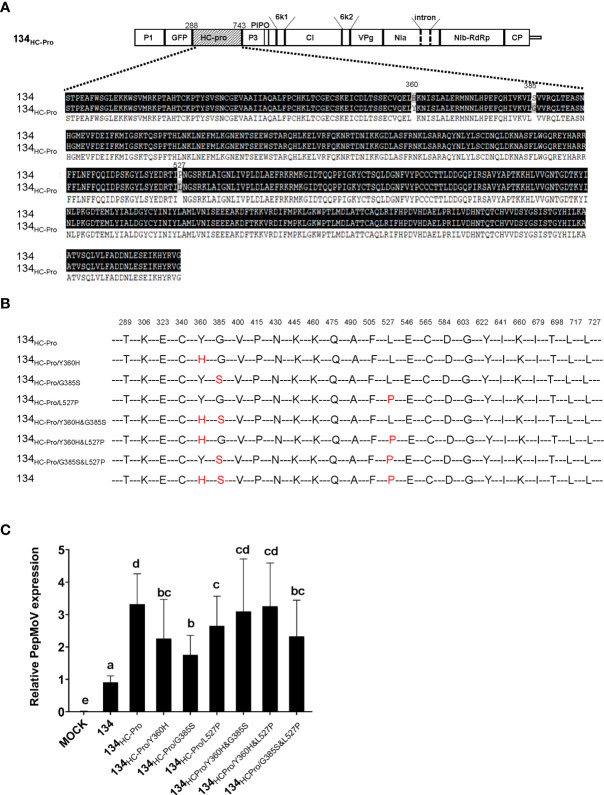
**(A)** Amino acid alignment sequence of the HC-Pro of PepMoV isolates 134 and 134_HC-Pro_. Dark highlighted amino acids are identical. Numbers are amino acid positions on the poly-protein precursor. **(B)** Schematic of PepMoV isolates 134_HC-Pro_-derived amino acid substitution mutants. Single or double amino acid substitution mutations (red) were introduced in 134_HC-Pro_. The positions of substituted amino acids on the polyprotein precursor are indicated at the top of the amino acid sequence. **(C)** Quantification of 134_HC-Pro_-derived mutants accumulation at 2 dpi. Plant tissues were collected from inoculated leaves at 2 dpi. Letters indicate a significant difference among 134, 134_HC-Pro_ and derived mutants according to a one-way ANOVA and Duncan’s least significant range test (*P* < 0.05).

In **Figure 2**, 134_HC-Pro_ and 134_NIa_ infected plants showed more severe necrotic symptoms when compared with 134. 134_HC-Pro_-infected plants showed more significant viral accumulation and faster movement at the early infection stage. An amino acid sequence alignment was conducted to determine the crucial domain of HC-Pro in PepMoV. Sequence comparison indicated that HC-Pro contains only three different amino acids (**Figure 5A**). We generated single and double amino acids substitution mutants to identify the crucial amino acid in 134_HC-Pro,_ which is responsible for faster virus accumulation and movement. The six 134_HC-Pro_-derived mutants were generated by adjusting the amino acid positions 360, 385, and 527 in PepMoV amino acid sequences and named 134_HC-Pro/Y360H_, 134_HC-Pro/G385S_, 134_HC-Pro/L527P_, 134_HC-Pro/Y360H&G385S_, 134_HC-Pro/Y360H&L527P_, and 134_HC-Pro/G385S&L527P_ (**Figure 5B**). We inoculated the plant by agro-infiltration and observed the GFP expression of all site-directed mutants. All plants exhibited similar GFP intensity at 2 and 3 dpi compared to 134 (**Supplementary Figure 3**). Therefore, we conducted RT-qPCR to quantify and compare viral RNA accumulation among these six mutants (**Figure 5C**). The results showed that all three single amino acid substitution mutants showed reduced accumulation levels compared to 134_HC-Pro_ but maintained significantly increased viral RNA accumulation compared to the 134. In addition, 134_HC-Pro/Y360H&G385S_, 134_HC-Pro/Y360H&L527P_, and 134_HC-Pro/G385S&L527P_, which are only one amino acid changed from 134, displayed higher RNA accumulation than 134 in **Figure 5C**, suggesting that each amino acid substitution (H360Y, S385G, and P527L) in 134 can increase the viral RNA accumulation.

### Amino acid substitution alters symptom development of PepMoV-134_NIb-CP_

**Figure 6:**
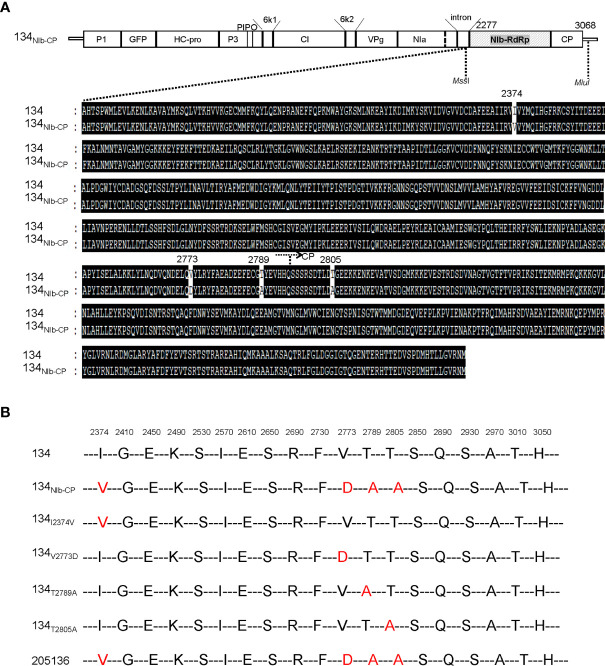
**(A)** Alignment of the deduced amino acid sequence of the NIb and CP of PepMoV isolates 134 and 205136. Dark highlighted amino acids are identical. Numbers are amino acid positions on the poly-protein precursor. **(B)** Schematic of PepMoV isolates 134-derived amino acid substitution mutants. The positions of substituted amino acids on the polyprotein precursor are indicated at the top of the amino acid sequence.

We showed that 134_NIb-CP_ induced weak viral symptoms and lower viral RNA accumulation than 134 (**Figure 2**). We assumed that the switched NIb and CP region changed severe symptoms caused by isolate 134 to mild symptoms caused by isolate 205136. To confirm whether the symptom differences between isolate 134 and 205136 might relate to this NIb-RdRp and CP region, we compared the amino acid sequence of the corresponding regions of isolate 134 and 205136 (**Figure 6A**). The amino acid differences are located at positions 2,374, 2,773, 2,789, and 2,805, three amino acids located in the NIb protein region, and the other is in the N-terminal of CP (**Figure 6A**). To identify crucial amino acid(s) among these four amino acids in the NIb and CP for symptom development, we constructed four 134_NIb-CP_-derived mutants which contained a single amino acid mutation (**Figure 6B**). The amino acid substitution mutants were named 134_I2374V_, 134_V2773D_, 134_T2789A_, and 134_T2805A,_ respectively.

**Figure 7:**
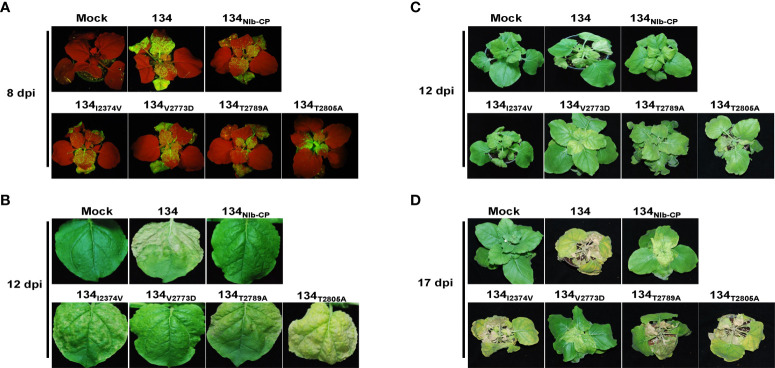
Virus replication and symptom development of 134 and substituted mutants on local and systemic leaves. PepMoV-134 and substituted mutants were inoculated onto *N. benthamiana* plants by agro-infiltration. The plants age used for inoculation is around 3 weeks old. The viral RNA accumulation difference among 134 and mutant viruses was monitored obviously for 5 days post-inoculation. **(A)** Observation of GFP expression on inoculated leaves at 8 dpi. **(B)** Results from systemic leaves at 12 dpi. Symptoms development of 134 and substituted mutants at 12 dpi **(C)** and 17 dpi **(D)**.

To compare infectivity and symptom development among the amino acid substitution mutants and 134, we inoculated the amino acid substitution mutants onto *N. benthamiana* plants by agro-infiltration. All four pPepMoV : GFP-134-derived single amino acid substitution mutants, 134_I2374V_, 134_V2773D_, 134_T2789A_, and 134_T2805A_, GFP-expressing virus existed on systemic leaves. Among them, the GFP intensity of the 134_T2805A_-infected plant was stronger than 134_NIb-CP_ and the other site-directed mutants (**Figure 7A**). 134_I2374V_, 134_T2789A_, and 134_T2805A_ displayed necrotic symptoms on systemic leaves at 12 dpi (**Figure 7**, panels B and C). The result indicated that the amino acid substitution of valine to aspartic acid at position 2773 is sufficient for PepMoV 134 to alter symptom development. When we incubated infected plants longer, 134_I2374V_, 134_T2789A_, and 134_T2805A_ showed more severe necrosis symptoms. However, 134_V2773D_ and 134_NIb-CP_ did not appear with severe necrotic symptoms at 17dpi (**Figure 7D**). Taken together, the crucial residue valine at position 2773 of NIb plays an essential role in PepMoV-derived symptom development.

## Discussion

The previous study described that PepMoV isolate 134 causes necrosis and yellowing, while 205136 causes severe mottle and yellowing symptoms in *N. benthamiana* plants (Kim et al., 2009). Here, we demonstrated that the 134_NIb-CP_ caused a more significant reduction in virus accumulation and mild symptoms than 134, suggesting that the C-terminal part of NIb is involved in PepMoV symptomatology and multiplication. In contrast, the 134_HC-Pro_ showed faster virus movement and increased severity indicating that HC-Pro plays an essential role in PepMoV symptom severity and systemic movement (**Figure 3**). To determine the crucial domain, we compared HC-Pro and NIb-CP amino acid sequences of 134 with counterparts of 134_HC-Pro_ and 134_NIb-CP,_ respectively, and constructed single/double amino acid substitution mutants (**Figures 5**, **6**). As a result, we showed that the amino acid residues tyrosine, glycine, and leucine at position 360, 385, and 527 of HC-Pro and 2773 positions of NIb might be involved in virus systemic movement and symptom development, respectively.

Because the HC-Pro protein of 134_HC-Pro_ was replaced from mild isolate 205136, theoretically, it could induce weaker symptoms and fewer virus accumulations. However, 134_HC-Pro_ caused more vigorous virus multiplication and faster long-distance movement than 134. It has been reported that the HC-Pro in the sugarcane mosaic virus and TuMV act as a viral RNA silencing suppressor (VSR; Han et al., 2016; Chen et al., 2017). The C-terminal HC-Pro protein in PVY also suppresses RNA silencing-based plant defenses by binding to small interfering RNAs. We were curious if HC-Pro of PepMoV 134 has a strong VSR activity than 205136. However, we did not compare the VSR activities of HC-Pro from PepMoV isolate 134 and 205136 in this study and needs additional works. In addition, one of the previous studies said that substituting Ile for Arg at position 180 in the conserved motif Phe-Arg-Asn-Lys (FRNK) of potyviruses contributed to symptom expression (Gal-On, 2000). The sequence FRNK is a conserved motif in 18 potyviruses and many different isolates of those viruses (Gal-On, 2000). The mutation of the FRNK box to FINK (R180I) causes a drastic reduction in symptom severity of the leaves of various cucurbit species without noticeably affecting virus accumulation or infectivity. This mutation has been exploited for use in cross-protection (Shiboleth et al., 2007). PepMoV isolates134 and 205136 have conserved FRNK motifs and have identical amino acid sequences. Therefore, the Arg of the FRNK motif is not the primary reason for the symptomatic difference between PepMoV isolate 134 and 205136. In this study, we found another three essential amino acid positions in HC-Pro of PepMoV, which affected viral accumulation, systemic movement, and symptom determinants in PepMoV isolate 134.

We showed that the 134_NIb-CP_ and 134_V2773D_ could not reproduce the necrotic symptom induced by the 134, although titer in systemic upper leaves was reduced relative to 134. Our results suggest that the C-terminal part of NIb is involved in PepMoV virus symptomatology and multiplication. Like the NIb protein of PepMoV, the *Oilseed rape mosaic virus* belongs to the genus *Tobamovirus*, in which RNA replicase functions in virus accumulation and disease symptoms determination. This appears to be the case of determinants of systemic necrotic spots in tobacco (Mansilla et al., 2009). Virus’ viral accumulation and symptomatology determinants are variable, depending on the specific virus family or strain. The symptoms and pathogenicity of different PepMoV isolates infect various plant hosts (Kim et al., 2009). Further work is required to identify crucial genes or motifs in PepMoV that might be involved in their symptoms and pathogenicity variation among the different plant hosts and virus isolates.

The 134_HC-Pro_ and 134_CI-NIa_, which substituted the HC-Pro region and half of the CI : NIa region replaced by counterpart regions of 205136, respectively, induced more robust virus distribution on the local leaves and faster long-distance movement on the systemic leaves. On the other hand, 134_HC-Pro_ showed distinctly more severe symptoms than 134, indicating that the HC-Pro protein of PepMoV roles in pathogenicity, virus accumulation, and systemic movement. Since we designed a chimeric construct based on the available restriction enzyme sites on pPepMoV : GFP-134 and 205136 isolate cDNA sequences, switched region in 134_CI-NIa_ construct included half of CI, 6K2, VPg, and N-terminal of NIa. Like 134_HC-Pro_, the 134_CI-NIa_ performed faster and stronger virus accumulation at 5 and 8 dpi (**Figures 3A, B**); however, it did not display severe necrosis symptoms earlier than 134 (**Figure 2A**). Based on the virus development process in plants, the RT-qPCR (**Figure 3C**) showed quantitative viral accumulation differences in the initial stage, GFP observation (**Figures 3A, B**) monitored virus cell-to-cell and systemic movement differences showed at the middle development stage, symptom observation (**Figure 2A**) displayed symptom differentiation at the late stage. In **Figure 2A**, the images of 134_NIa_ were taken at 12 dpi; it is the late stage of the PepMoV infection, and 134_NIa_ caused much more severe and accelerated necrotic symptoms than 134. However, in **Figures 3A, B**, images of 134_NIa_ were taken at 5 and 8 dpi, respectively, at the early infection stage (5 dpi), GFP fluorescence expression did not show a clear difference compared to 134. However, 134_NIa_ developed its symptom much faster than 134 after 5 dpi; they already started to show necrosis symptoms at 8 dpi (data not shown). Additionally, in **Figures 2A, B**, because the objectives of the two experiments were different, we experimented using two different inoculation methods, i.e., agro-infiltration and sap inoculation (saps were collected from infected leaves), respectively. Meanwhile, the symptom images in **Figures 2A, B** were taken at 12 dpi and 8 dpi, respectively. As a result, the degree of 134_HC-Pro_ and 134_NIa_-induced necrosis in **Figures 2A, B** was slightly different. The NIa from PepMoV has been demonstrated to determine pathogenicity (Gong et al., 2020). The result of this study said that 134_NIa_ seems to have reached a similar conclusion with them.

Further, 134_NIa_, generated by modifying the counterpart of NIa C-terminal between isolates 134 and 205136, contains 177 amino acids, and only two are different (**Supplementary Figure 4**). This suggests that one of these amino acids may play an essential role in the pathogenicity factor (data unavailable). Even though we could not determine the specific proteins or motifs involved in more robust virus accumulation and faster virus systemic movement in 134_CI-NIa_, further detailed experiments may clarify the specific roles of PepMoV viral proteins, whose functions remain unclear. This study may help to understand pathogenic determinant(s) in the PepMoV-*N. benthamiana* pathosystem and increase the understanding of the different host responses to PepMoV isolates.

## Data availability statement

The original contributions presented in the study are included in the article/**Supplementary Material**. Further inquiries can be directed to the corresponding authors.

## Author contributions

MF, JY, H-RK, and K-HK designed the project. MF performed the experiments. MF, JY, H-RK, and K-HK analyzed the data. MF wrote the draft manuscript with editorial contributions from JY and K-HK. K-HK supervised the study and funding. All authors contributed to the article and approved the submitted version.

## Funding

This research was supported in part by grants from the Korea Institute of Planning and Evaluation for Technology in Food, Agriculture, and Forestry (320037-05-1-HD020), funded by the Ministry of Agriculture, Food and Rural Affairs, and from the Agenda Program (PJ01488703), Rural Development Administration, Republic of Korea. MF was supported by a Brain Korea 21 Plus Project research fellowship.

## Acknowledgments

We would like to acknowledge the contribution of Dr. Phu-Tri Tran.

## Conflict of interest

The authors declare that the research was conducted without any commercial or financial relationships construed as a potential conflict of interest.

## Publisher’s note

All claims expressed in this article are solely those of the authors and do not necessarily represent those of their affiliated organizations, or those of the publisher, the editors and the reviewers. Any product that may be evaluated in this article, or claim that may be made by its manufacturer, is not guaranteed or endorsed by the publisher.
