# Supplementary material for: Identification of viral genes involved in pepper mottle virus replication and symptom development in Nicotiana benthamiana
Source: Front Plant Sci. 2022 Oct 28;13:1048074. doi: 10.3389/fpls.2022.1048074 (PMC9650420; doi:10.3389/fpls.2022.1048074)
Supplement: Supplementary file 1 [file DataSheet_1.pdf]

Supplementary Material

1.1 Supplementary Figures

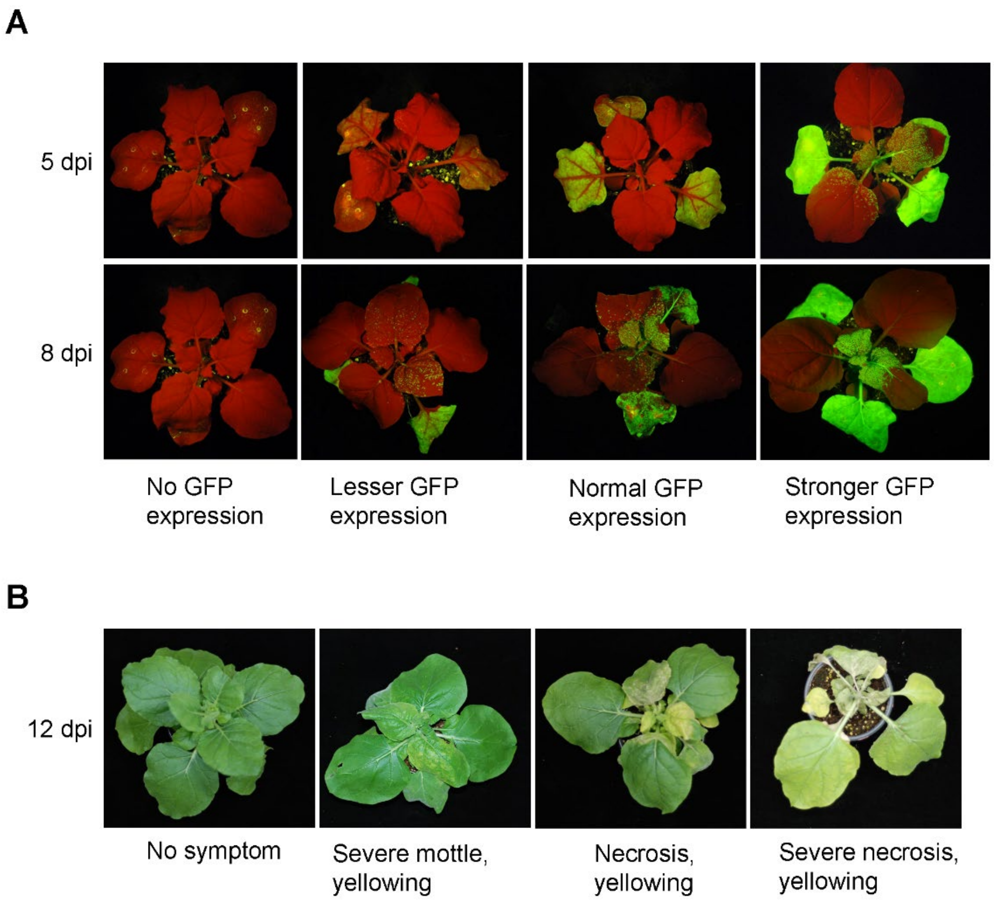

Supplementary Figure 1. (A) Scale card of GFP fluorescence expression. (B) Scale card of symptom expression.

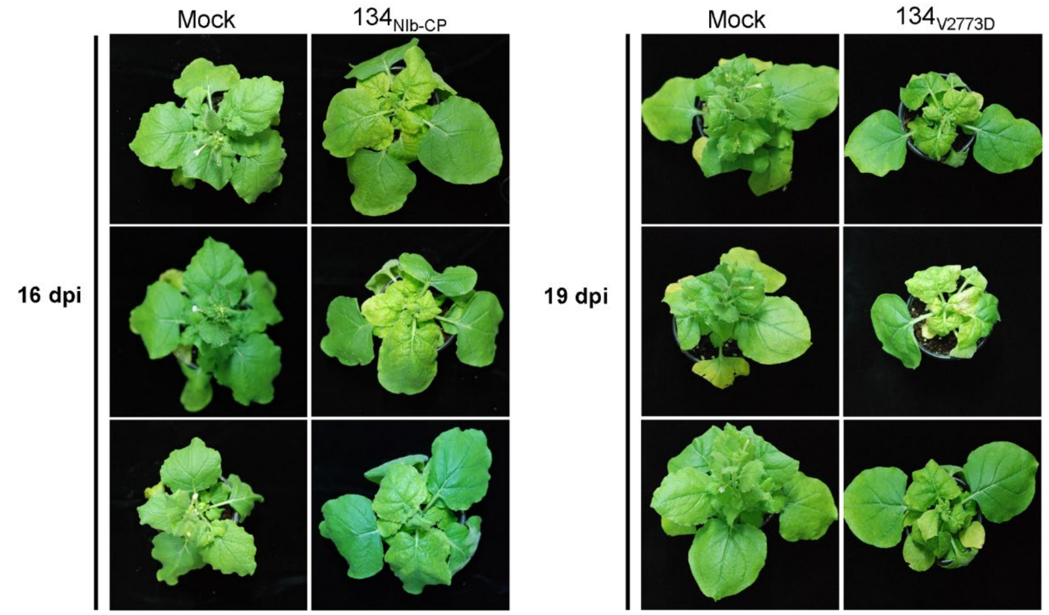

Supplementary Figure 2. Symptom development of $134_{NIb\text{-}CP}$ and 134_{V2773D} were monitored extra few days to confirm no necrotic symptoms. (A) Symptoms of mock and $134_{NIb\text{-}CP}$ at 16 dpi. (B) Symptoms of mock and 134_{V2773D} at 19 dpi.

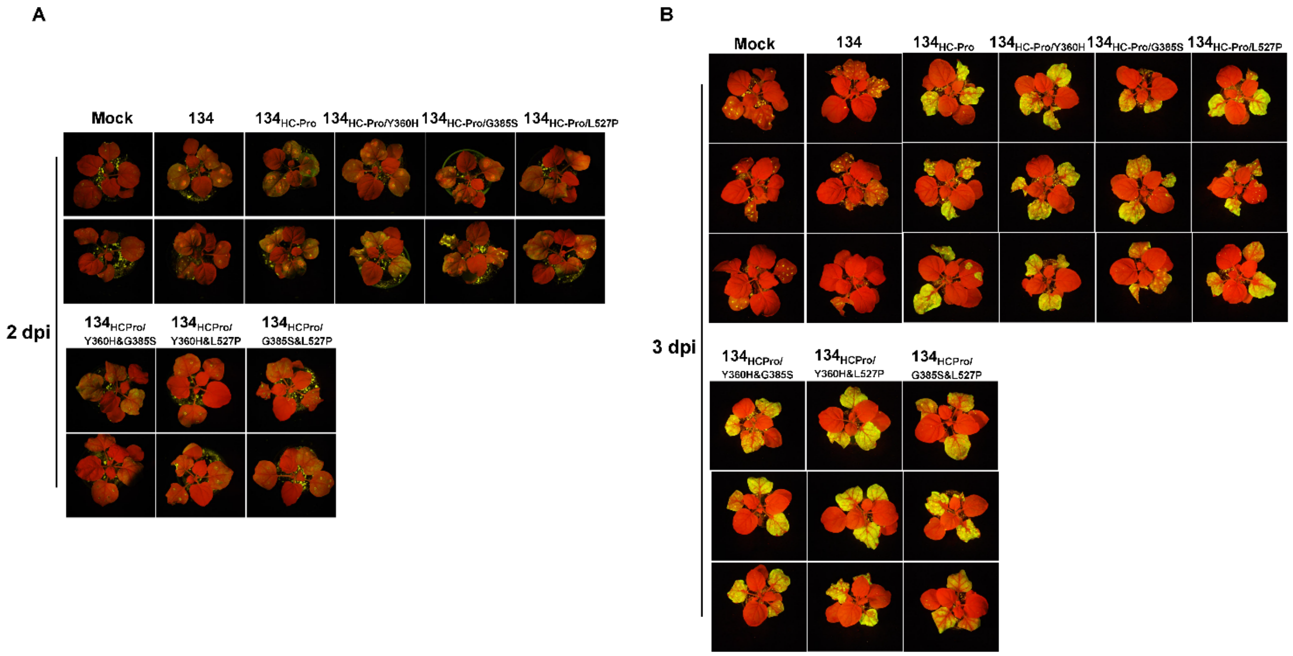

Supplementary Figure 3. Observation of GFP expression of 134 and $134_{HC\text{-}Pro}$-derived mutants after agroinfiltration on *N. benthamiana*. GFP fluorescence representing virus infection on plants at 2 dpi (A) and 3 dpi (B).

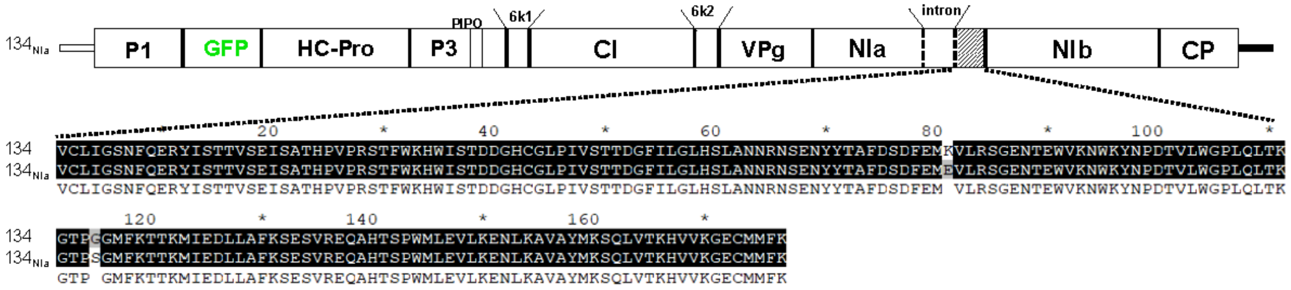

Supplementary Figure 4. Amino acid alignment sequence of the C-terminal NIa of PepMoV isolates 134 and 134_{NIa}.

1.2 Supplementary Table

Primer name	Sequence 5'-3'	Restriction enzymes	Purpose
pCAMBIA 6601 Fw	GCTGGCTGGTGGCAGGATA	*Bsp*120I<br>*Acc*65I	Construction of 134_{P1}
P1 *Kpn*I Rv	GATGAACGTTAACGGTACCCGAATACTG TTCCATATGAAGTACAGTTGC		
PepMoV 948 Fw	GTTCGCGGAAAATCGGATGG	*Acc*65I<br>*Pac*I	Construction of $134_{\text{HC-Pro}}$
PepMoV 2511 Rv	ACATGACACTTGGCCTGTAGATCCC		
PepMoV 2222 Fw	TGACGCAGAGCTGCCTCGTAT	*Pac*I<br>*Sac*I	Construction of $134_{\text{P3-CI}}$
PepMoV 4727 Rv	TGCGGTGCCCTTCTGTATGCG		
PepMoV 3940 Fw	ACGGCAGGGTTTTGCTGATA	*Sac*I<br>*Mss*I	Construction of $134_{\text{CI-NIa}}$
PepMoV NIa-Intron2 ST-LS1 Fw	AACAGACAGGTTTGTTTCTGCTTCTACCT TTGAT		
Intron2 ST-LS1-PepMoV NIa Rv	GAGACACACCCTAAACATCACCATGTTTT GGTCA		Construction of 134_{NIa}
PepMoV 7262 Rv	TGCGTCGCAATCGACTACTCCT		
PepMoV 6988 Fw	GGGAGCAAGCACACACATCACCT	*Mss*I<br>*Mlu*I	Construction of $134_{\text{NIb-CP}}$
pSNU1 241 Rv	TCGCAAGACCGGCAACAGGA		
mutant4 I2374V Fw	TATCATTAGAGTTGTTGTATACATGCAGA	*Mss*I<br>*Mlu*I	Construction of 134_{I2374V}
mutant4 I2374V Rv	TCTGCATGTATACAACAACTCTAATGATA		
mutant4 V2773D Fw	GAGCTTCAGGACTACCTCAGA	*Mss*I<br>*Mlu*I	Construction of 134_{V2773D}
mutant4 V2773D Rv	TCTGAGGTAGTCCTGAAGCTC		
mutant4 T2789A Fw	TTGAATGTGGTGCATATGAAGTTCA	*Mss*I<br>*Mlu*I	Construction of 134_{T2789A}
mutant 4 T2789A Rv	TGAACTTCATATGCACCACATTCAA		
mutant4 T2805A Fw	GATACATTGGATGCTGGAGAGGAGA	*Mss*I<br>*Mlu*I	Construction of 134_{T2805A}
mutant4 T2805A Rv	TCTCCTCTCCAGCATCCAATGTATC		
PepMoV diag Fw	ATGAGCAGCTCAAGATCAGATACATTG		PepMoV detection primer
PepMoV diag Rv	CATATTCCTGACCCCAAGCA		PepMoV detection primer
PepMoV 5836 Fw	GCACAACCGTTGGCATGGGC		PepMoV quantification in RT-qPCR
PepMoV 5950 Rv	TCCATTTGTGCACCTGTGAGTGG		PepMoV quantification in RT-qPCR
Nb Actin real time Fw	CCAGGTATTGCTGATAGAATGAG		Internal control for RT-qPCR

Nb Actin real time Rv	CTGAGGGAAGCCAAGATAGAG		Internal control for RT-qPCR

Supplementary Table 1. Primers and restriction enzymes used in this study.
